# Telemedicine and Remote Monitoring as an Adjunct to Medical Management of Bronchiectasis

**DOI:** 10.3390/life11111196

**Published:** 2021-11-06

**Authors:** Soontharee Congrete, Mark L. Metersky

**Affiliations:** Division of Pulmonary, Critical Care and Sleep Medicine, University of Connecticut School of Medicine, Farmington, CT 06032, USA; congrete@uchc.edu

**Keywords:** bronchiectasis, remote monitoring, telemedicine, telehealth

## Abstract

The limited resources and the practice of social distancing during the COVID pandemic create a paradigm shift in the utilization of telemedicine in healthcare. However, the implementation of best practices is hampered in part by a lack of literature devoted to telehealth in bronchiectasis. In this commentary, we examine multiple approaches to structuring of telemedicine care for patients with bronchiectasis, highlight current evidence-based interventions that can be incorporated into the management of bronchiectasis, and describe our experience with telemedicine at the University of Connecticut Center for Bronchiectasis Care during the COVID-19 pandemic. The structural model must be adapted to different local dynamics and available technologies with careful attention to patient characteristics and access to technology to avoid the potential paradoxical effects of increasing patients’ burden and healthcare disparities in underserved populations.

## 1. Introduction

Bronchiectasis is a chronic respiratory disease characterized by permanent abnormal dilatation of the bronchi, driven by chronic airway inflammation. Bacterial infection is a cornerstone in the development and progression of bronchiectasis. Cole’s vicious cycle hypothesis [1] illustrates how airway dysfunction, airway inflammation, infection, and structural damage are connected. An environmental insult, often on a background of genetic susceptibility and impaired mucociliary clearance results in the persistence of microbes in the bronchial tree. Microbial infection results in chronic inflammation, tissue damage, and impaired mucociliary motility. This leads to a cycle of progressive inflammation and airway lung damage. In this model of disease, exacerbations work as a catalyst, accelerating the progression of the disease [2,3]. Prevention, early recognition, and treatment of exacerbations may avert poor outcomes. Patients with three or more exacerbations per year having double the mortality of those who do not experience exacerbations (hazard ratio, 2.03; 95% confidence interval, 1.02–4.03) [4]. Exacerbations of bronchiectasis are associated with increased airway and systemic inflammation [5] and progressive lung damage [6]. More severe and more frequent exacerbations are also associated with worse quality of life [7] and accelerated lung function decline [8]. In addition, the economic burden of this disease increases with disease severity, hospitalizations, need for intensive care, and use of inhaled antibiotics [9,10]. Consequently, exacerbations of bronchiectasis are key targets for therapy [11].

Many patients with bronchiectasis receive care at specialized centers, often far from their homes. This limits the ability of patients to be closely monitored or to receive care quickly for changes in their status, including exacerbations. Telemedicine is an attractive tool that has the potential to improve care for patients with bronchiectasis. It allows physicians to remotely monitor patients and quickly intervene when patients deteriorate. Telemedicine also provides the opportunity for answering patient queries in an efficient manner, using fewer resources than required for a face-to-face visit. 

Bronchiectasis is a disease with high economic burden, especially related to exacerbations [12]. Over 110,000 persons in the US may be receiving treatment for bronchiectasis, resulting in medical-care expenditures of USD 630 million annually [10]. In the US, a total cost of USD 26,000 is reported in patients without exacerbations, increasing to USD 36,000–37,000 in patients with exacerbations [13]. A Spanish study reported a higher total annual cost for patients with >2 exacerbations per year (EUR 7520) compared with those without exacerbations (EUR 3892) [14]. Although there is no current cost–benefit analysis for the utilization of telemedicine in bronchiectasis, some studies in patients with COPD demonstrated reduced costs among the telemedicine group [15,16,17]. Paré G et al. found that telemonitoring over a 6-month period generated USD 355 in savings per patient, or a net gain of 15% compared to traditional home care in patients with COPD [18]. A study demonstrated remote spirometry could result in financial savings of EUR 36,802–40,397 per patient over 10 years in patients with cystic fibrosis compared to the control groups who received only ambulatory visits [19] Another study demonstrated that telemedicine is more cost effective (USD 335/patient/year) as compared to patient travel (USD 585/patient/year) and provision of onsite subspecialty care (USD 1166/patient/year) [20]. Although cost effectiveness depends on the local healthcare reimbursement system, it is likely that by reducing the number of in person patient consultations, possibly, there would be a reduced cost for patients’ care as a result of exacerbations and hospitalizations [21]. These data have implications for time and cost saving for both patients and the healthcare system with the utilization of telemedicine in bronchiectasis.

In this commentary, we discuss how telemedicine can be an effective tool for exacerbation prevention and management and routine monitoring of stable patients, possibly leading to a reduction in healthcare costs and improved healthcare access. In addition to reviewing the relevant literature, we cite observations from our telemedicine experience at the University of Connecticut Center for Bronchiectasis Care during the COVID-19 pandemic.

## 2. Home Monitoring of Treatment 

Telemedicine has been used for the delivery of routine appointments, annual assessments, and adherence monitoring for many chronic health conditions. While there is little literature devoted to telehealth in bronchiectasis, there has been much experience with telehealth in cystic fibrosis (CF), and much of that experience is relevant to non-CF bronchiectasis. Telehealth has been used to improve access to healthcare for CF patients, as they often require multi-disciplinary consultation and referral to CF centers, a long distance from where they live [22]. Telehealth has the potential to be particularly impactful for patients in remote or low-income settings, who often have a poor understanding of the disease and poorer health outcomes [23]. The ease of wireless healthcare may lead to its greater penetration into these populations, helping to improve health disparities. However, lack of access to a high-speed internet service or state-of-the-art devices among these populations could paradoxically worsen health disparities. Therefore, the impact of telehealth will need to be carefully monitored to avoid unanticipated adverse consequences.

Common parameters used to monitor disease progression and exacerbations include clinical signs and symptoms, lung function, imaging, and sputum cultures. These data can be simply obtained or processed via teleconferences and electronic medical record systems. The patients can report pulmonary symptoms, sputum color, volume or viscosity, levels of physical activity, health-related quality of life (QoL) questionnaires, and weight changes. Strategies including text messaging reminders and web-based and mobile applications to monitor and record symptoms can be helpful [24].

Vital signs and oxygen levels can be collected by the patients and/or the family with affordable commercial devices (sphygmomanometer, thermometer, pulse oximetry) at rest and with exertion. Cardiopulmonary parameters can also be acquired from wearable accelerometer-based biosensors [25]. Furthermore, home spirometry can be considered to measure lung function in bronchiectasis [26].

These data can be used to identify exacerbations, thereby creating opportunities for preventive interventions. The two bronchiectasis disease severity measures, the Bronchiectasis Severity Index (BSI) and FACED predict severity and mortality, both include FEV1, dyspnea, *Pseudomonas* colonization, and radiographic features. BSI additionally includes prior exacerbation history and predicts morbidity, exacerbations, and hospitalization rates [4,27]. Based on these scoring systems, preventive interventions such as aggressive airway clearance therapy or the initiation of a self-treatment plan with medications can be instituted. In addition, these severity scoring tools may be useful in tailoring the frequency of routine monitoring.

Telemedicine also has a potential role in monitoring therapeutic interventions. An ongoing randomized controlled trial, VIRTUAL-CF, is using remote spirometry and oxygen saturation measurements to monitor patients with CF receiving intravenous antibiotics in the community and assessing whether a multidisciplinary teleconference improves health-related quality of life as compared to the standard face to face visit [28]. Furthermore, comprehensive information collected on health status and treatment can be used to develop personalized interventions that influence a positive health impact, such as establishing appropriate inhaler use. 

Among patients with chronic diseases, poor medication adherence is associated with worse outcomes, unnecessary escalation of treatment, and increased healthcare utilization. Besides data collection to understand the reasons for non-adherence such as medication side effects, digital interventions to improve adherence such as electronic inhalers, text messaging, and reminders have been used. Devices with an audiovisual reminder function can significantly improve adherence with inhaled corticosteroid therapy in adult asthma [29]. The I-Neb adaptive aerosol delivery (AAD) nebulizer device (used for CF and bronchiectasis) adapts medication delivery to the patient’s breathing pattern and provides real-time audiovisual feedback [30]. The device can also be used to monitor the frequency and length of treatments. Adherence to the treatment was maintained between 60% and 70% over 1 year [31]. Insight Online is a home monitoring telehealth interface, which includes data from the I-Neb. Treatment goals and progression can be tracked by the patients. Data showed that the patients who did engage had higher adherence; however, over 50% of the participants failed to upload regularly. This result raises the issue of increased treatment burden on patients and the possibility that some patients may find this level of monitoring intrusive [32].

Videoconference is a good platform to improve patients’ education for airway mucous clearance techniques (i.e., oscillating PEP devices, manual techniques, and postural drainage), which are the cornerstone of therapy for patients with bronchiectasis as well as the active cycle of breathing techniques (ACBT). The physicians can provide real-time feedback to the patients with home devices (i.e., vest therapy, flutter valve) as well as the online instructions of the devices and techniques. Similar strategies can also be used to improve education on nebulizer machines for saline solutions and bronchodilators. 

At the University of Connecticut Center for Bronchiectasis Care, we relied heavily on telehealth visits for bronchiectasis patients during the COVID-19 pandemic. Patients valued the ability to discuss their concerns and undergo monitoring, while avoiding the potential risk of interacting with others at a healthcare facility. We found it relatively easy and effective to treat mild exacerbations and regularly monitor for side effects of our patients undergoing treatment for non-tuberculous mycobacterial infection. Among our more than 125 bronchiectasis and non-tuberculous mycobacteria patients, to our knowledge, only one was admitted to the hospital for an exacerbation of bronchiectasis during the pandemic, suggesting that the care has been effective, at least in preventing severe exacerbations.

## 3. Home Monitoring for Early Detection of Exacerbation

Bronchiectasis exacerbations cause substantial morbidity and mortality [33]. Longitudinal studies have described up to a 30% mortality at 1-year follow-up after a severe exacerbation [34,35] as well as a 46% readmission rate [36]. In two studies, after admission to the ICU due to exacerbation, the mortality rate was 40% at one year [37] and 60% at four years [38]. 

Common parameters and various home devices used to monitor disease progression and exacerbation through telehealth have been mentioned above. A study assessing the use of smartphone applications for the earlier recognition of exacerbations in adults with CF through changes in respiratory symptoms demonstrated more rapid detection of exacerbations that required antibiotics; however, it did not demonstrate a clear effect on the number of courses of IV antibiotics, lung function (FEV1), or health-related quality of life [39]. FVC and FEV1 are significantly reduced during acute exacerbations and recover during convalescence [40]. These can be measured by home spirometry, which may be useful in patients who have vague symptoms. 

Overall, the relevant data can be summarized, as demonstrating that remote monitoring may be able to help identify exacerbations of CF (and by extrapolation, bronchiectasis), but there is no convincing data that doing so improves overall patient outcomes.

## 4. Lung Function Monitoring with Home Spirometry and Peak Expiratory Flow Rate (PEFR)

Lung function deterioration is a major cause of morbidity and mortality in patients with CF [41] and to a lesser but significant extent for patients with bronchiectasis [42]. Lung function correlates with exacerbation rates and overall severity of disease, based on the BSI and FACED scoring systems. Routine monitoring of lung function may be of value for both stable patients and those with clinical deterioration. Worsening lung function should prompt consideration of more aggressive interventions, including more effective airway clearance or chronic antibiotic treatment, including chronic macrolide therapy or inhaled antibiotics.

During an acute exacerbation, FVC and FEV1 decline significantly [40], as does as peak expiratory flow rate (PEFR), a simple and well-established tool for assessing daily changes in lung function. At exacerbation, the mean PEFR dropped by 10.6% [43]. The peak flow meter is less complicated to use than home spirometry; however, caution is needed with interpretation, as PEFR is much more effort dependent than spirometry.

Emerging evidence supports the use of home spirometry in bronchiectasis. Among 77 stable CF patients, investigators found that home spirometric measures (FEV1, FVC, and PEFR) have strong correlations with the corresponding baseline lung function and concluded that home spirometry is a reliable device in monitoring CF patients [26]. In another cohort of 40 stable CF patients, researchers also found that home spirometry provides similar estimates of lung function (FEV1 and FVC) compared to standard in-clinic devices; however, PEFR tended to be higher on the home devices by 0.542 ± 1.12 L/s on average [44]. While these data seem promising to be applied to patients with non-CF bronchiectasis, future research is required to evaluate validation of home spirometry to office-based spirometry in patients with non-CF bronchiectasis and to determine if such monitoring can contribute to improved patient-centered outcomes.

**Figure 1:**
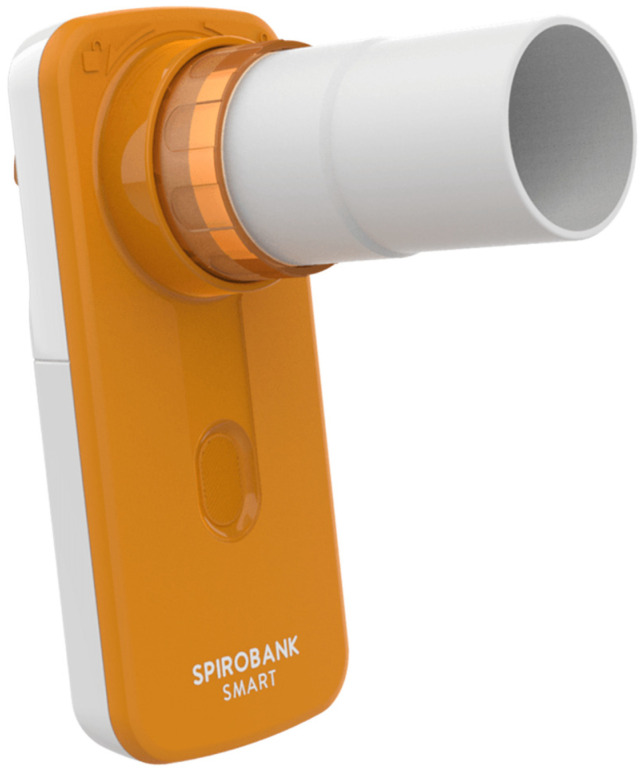
Home Spirometry (ZEPHYRx^®^ Spirobank Smart). (Retrieved 1 November 2021, from https://www.spirometry.com/approfondimenti/mirtogether-with-zephyrx-in-remote-patient-monitoring-and-spirometry-gaming).

Mobile-phone-based portable spirometry has been developed in recent years. SpiroSmart is a low-cost mobile phone application that performs spirometry using the built-in microphone with a mean error when compared to a clinical spirometer of 5.1% for common measures of lung function [45]. ZEPHYRx^®^ is a mobile-app-based, gamified spirometer connected to the mobile phone with which daily performance can be tracked. It can measure FVC, FEV1, PEFR, FEV1/FVC, FEV6, FEV25-75, and forced inspiratory vital capacity (FIVC) and can demonstrate flow volume loop and time–volume graphs. The validation of the device is being investigated in an NIH-funded randomized control trial (Figure 1) [46].

A small study showed promising results, measuring FEV1 with a telehealth program using data collected from a home spirometry device and sent to the CF center. The patients were contacted if they met intervention criteria for an exacerbation based on FEV1 decline or oxygen saturation. A slower decline in annual FEV1 was observed in the telehealth group [47]; however, this outcome was not found in a subsequent large multicenter randomized controlled trial. This study used twice weekly home spirometry and respiratory symptom scoring to trigger contact with CF patients after a reduction in FEV1 >10% or an increase in respiratory symptoms. Despite no significant finding in slowing FEV1 decline, the early intervention group had a shorter time to their first exacerbation and more exacerbation treatments compared with the usual care group, suggesting that telehealth intervention may be able to detect exacerbations earlier, prompting early treatment [48].

## 5. Home Monitoring of Physical Activity

Patients with bronchiectasis have decreased exercise tolerance as a result of impaired muscle function and decreased physical activity [49]. They commonly present with fat-free mass depletion, regardless of etiology, leading to further decline in exercise tolerance [50]. Higher levels of physical activity correlate with a better quality of life and higher aerobic capacity. In addition, highly active patients experience a slower decline in FEV1 [49]. Thus, the promotion of daily physical activity and exercise may be an important component of bronchiectasis management, especially for patients with more advanced disease and impaired pulmonary function.

Numerous tools in conjunction with telemedicine can be used to assess daily physical activity in patients with bronchiectasis. In addition to patient-reported data or the International Physical Activity Questionnaire (IPAQ), levels of physical activity can be quantified by fitness trackers, accelerometers (ActiGraph), pedometers, or mobile phones using steps trackers. ActiGraphy or pedometers were thought to be superior to IPAQ, as they measure the intensity of physical activity and yield a more precise measure of time spent walking. IPAQ did not appear to represent an accurate measure of physical activity in this population, in part due to the complexity of the tool [51]. While there are no current data in bronchiectasis, multiple studies reported that intervention programs that combine the use of wearable monitors (i.e., pedometers, accelerometers) with goal setting can increase daily physical activity, 6-minute walking distance (6MWD), Modified Medical Research Council Scale (mMRC), COPD assessment test (CAT) [52], and improved quality of life in patients with COPD [53,54,55].

## 6. Home Monitoring for Quality of Life (QoL)

Several tools have been validated to assess health-related quality of life (HRQOL) as a patient-centered outcome in patients with bronchiectasis. These can be incorporated into each telemedicine visit. The patients or the medical assistants can fill out the form in the electronic medical system before the encounter. The development of mobile-app-based remote monitoring may also be beneficial in the future. HRQoL is defined as “the perception of the impact of health on an individual’s contentment or satisfaction with life in areas they consider important” [56]. The most widely used are St George’s Respiratory Questionnaire (SGRQ), the Leicester Cough Questionnaire Score (LCQ), Quality of Life Questionnaire—Bronchiectasis (QOL-B), and the Bronchiectasis Health Questionnaire (BHQ). Despite differences in the construct of these questionnaires, there is good evidence to support their validity, internal reliability, and repeatability. The comparative validity of HRQOL questionnaires used to assess bronchiectasis has not been investigated. There was a stronger correlation between HRQOL and subjective outcome measures, such as dyspnea and fatigue, compared with objective measures, such as exercise capacity and extent of bronchiectasis on CT scan. This suggests that HRQOL questionnaires assess a unique aspect of health not captured by objective measures [57].

The Quality of Life Questionnaire—Bronchiectasis (QOL-B) was the first disease-specific, patient-reported outcome measure for patients with bronchiectasis, which contains 37 items on eight scales (respiratory symptoms, physical, role, emotional and social functioning, vitality, health perceptions, and treatment burden). It has demonstrated excellent psychometric properties including reliability and validity in two large-scale clinical trials [58].

**Figure 2:**
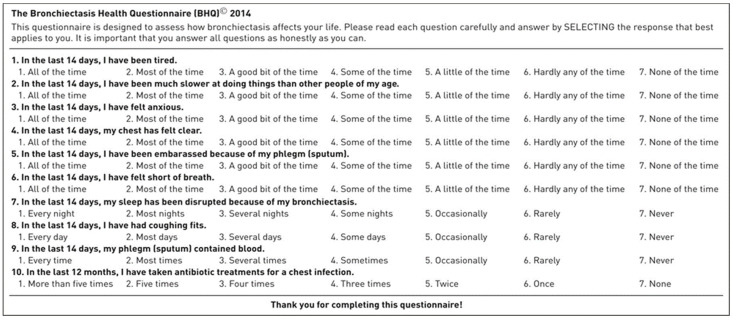
Bronchiectasis Health Questionnaire.

However, these questionnaires have limitations of being relatively long and lacking generation of a single total score (QOL-B) and are not disease-specific (SGRQ and LCQ). A publication validated the use of the BHQ score developed specifically for patients with bronchiectasis [59]. It consists of simple 10 items that generate a single total score and has good validity and repeatability (Figure 2). There was a significant association between BHQ scores and the number of exacerbations of bronchiectasis in the last 12 months, hospital admissions, and computed tomography scan bronchiectasis-involved pulmonary lobe counts. It can be used in the telemedicine visit to assess the impact of bronchiectasis from the patient’s perspective.

## 7. Tele-Rehabilitation

In patients with bronchiectasis, muscle weakness and physical inactivity may impair quality of life. International guidelines recommend pulmonary rehabilitation for individuals who are functionally limited by shortness of breath (Modified Medical Research Council (MMRC) Dyspnea Scale ≥1 [60].

Pulmonary rehabilitation increases exercise capacity, improves the quality of life in individuals with bronchiectasis, and may reduce the frequency of exacerbations; however, these effects were not maintained at 6 months [61,62].

The concern is whether pulmonary rehabilitation for bronchiectasis might increase healthcare costs without sustained improved outcomes. So, is it worthwhile? A pilot study identified no significant benefits associated with pulmonary rehabilitation after exacerbations of bronchiectasis [63]. However, the role of pulmonary rehabilitation after an acute exacerbation of bronchiectasis needs to be explored with larger studies. A study found that patients with bronchiectasis and COPD have similar adherence and completion rates of pulmonary rehabilitation and also have a similar magnitude of improvement in exercise capacity and health-related QOL to patients with matched-group COPD [64]. These data support emerging evidence supporting pulmonary rehabilitation in patients with bronchiectasis, since the beneficial effects of pulmonary rehabilitation in COPD are well established in clinical trials [65].

Despite the guidelines’ recommendations and emerging evidence, pulmonary rehabilitation is underutilized in patients with bronchiectasis [66]. There is a tremendous opportunity for tele-rehabilitation to improve utilization, as transportation barriers are a predictor of poor adherence to pulmonary rehabilitation attendance [67,68]. Application of telehealth in pulmonary rehabilitation may provide greater access and service delivery options for individuals who are geographically or socially isolated, for patients in full-time work or study, or for individuals who find travel difficult due to their disease severity or comorbidities [69]. A recent study investigated a home-based pulmonary rehabilitation (HBPR). The intervention was safe and well tolerated and provided short-term improvements in functional capacity, peripheral muscle strength, and quality of life in people with bronchiectasis. However, the program was not effective in maintaining improvements after a 6-month follow-up period [70]. Because many of the benefits of pulmonary rehabilitation require ongoing physical training after the acute rehabilitation program, tele-rehabilitation can potentially facilitate ongoing exercise and longer-term benefits.

## 8. Experience in Telemedicine in Patients with Bronchiectasis

During the COVID-19 pandemic, healthcare facilities have been adopting telemedicine to better allocate resources and prevent unnecessary exposure during essential outpatient visits [71]. Bronchiectasis is a complex chronic disease that requires long-term monitoring and often requires specialist consultations or referral to bronchiectasis centers. The advantage of telemedicine is the ability to increase patients’ access to healthcare, while avoiding travel and exposure to potentially infected individuals.

Based on a study of COPD patients’ experience of a telemedicine intervention, telemedicine brought enhanced wellbeing and a sense of security of being monitored and allowed appropriate interventions in case of changes in the patient’s condition. Patients also developed increased awareness and better self-management of their disease. They experienced more focused and less stressful meetings via video consultations than in typical visits [72]. At this time, there are no similar data for patients with bronchiectasis.

At the University of Connecticut, we found that telehealth was invaluable in facilitating care for patients reluctant to leave their homes during the COVID-19 pandemic. To maximize efficiency, before providers saw the patients, our nurses or medical assistants performed the same “rooming” functions, other than vital signs, as they did for face-to-face visits. These included reconciling medications, entering symptom scores when relevant, and determining if the patient had specific questions or concerns. They also attempted to verify that the patient was able to access the necessary software. We found that the technology was usually time efficient, although there was occasional difficulty among some patients using the software.

The inability to perform a physical examination, particularly lung auscultation, could impact the quality of care in some cases. However, this was not always the case; even with low-resolution cameras, there were times that examination was useful, with specific examples being the identification of new-onset edema and a drug rash. High-tech solutions that allow remote physical examination are available, although it is unrealistic to think that they will soon be deployed in a large-scale manner, given their cost. However, multiple tools can be used as a surrogate for physical assessment, as discussed above.

Telemedicine should only be considered when the patients have computers, smartphones, or tablets supporting the software and give consent to the telehealth visits. Patients who reside a long distance from their provider or have mobility limitations, those being quarantined for COVID-19 exposure, or require intensive monitoring are the most likely to benefit from tele-visits. Patients who have an auditory impairment or intellectual disability may not be candidates for telehealth visits unless they are accompanied by family or caretakers who can assist with the visit. All of these factors should be considered when determining which patients may be appropriate for telehealth visits.

**Figure 3:**
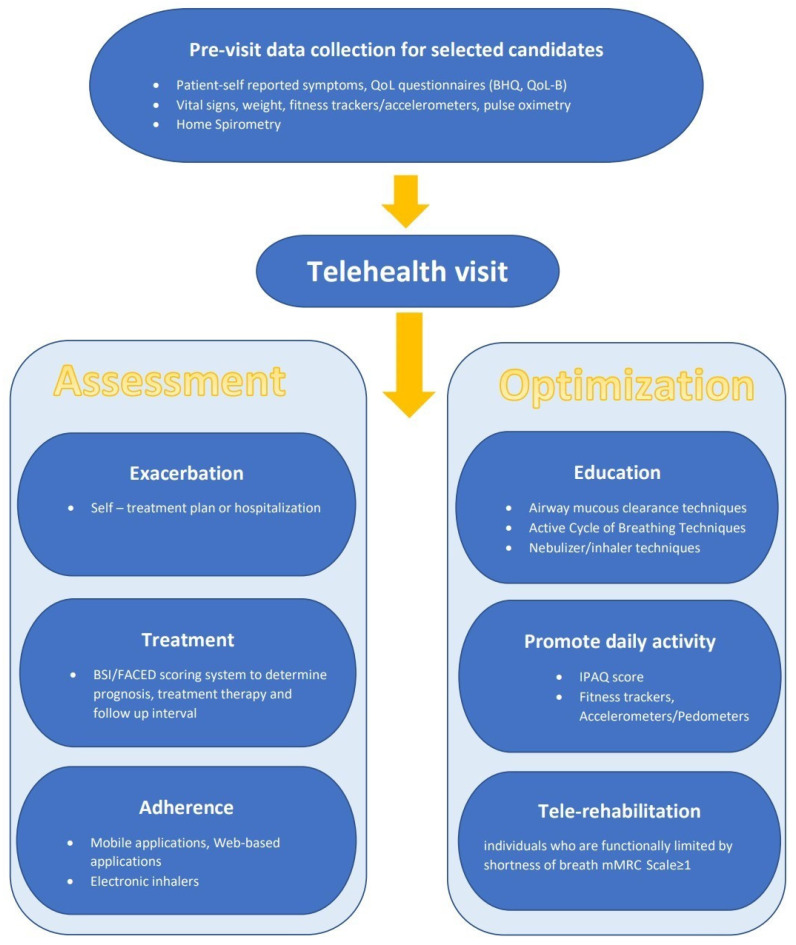
Potential components of a telehealth visit for patients with bronchiectasis.

In summary, the expanded availability of digital health technologies has created the prospect of enhancing comprehensive care and healthcare access in patients with bronchiectasis and has been particularly valuable during the COVID pandemic. The structural model of telemedicine can be established based on current evidence and tailored to each individual based on the availability of the necessary technologies; we provide a suggested framework in Figure 3. However, limitations should be carefully considered, and future research is needed to explore some areas of uncertainty. These include the role of monitoring technology, tele-rehabilitation, and identification of patients not appropriate for telemedicine. As the use of telemedicine inevitably will increase, there needs to be careful attention so that its use does not promote healthcare disparities among medically underserved populations.

## Data Availability

Exclude.
